# MatchingLand, geospatial data testbed for the assessment of matching methods

**DOI:** 10.1038/sdata.2017.180

**Published:** 2017-12-05

**Authors:** Emerson M. A. Xavier, Francisco J. Ariza-López, Manuel A. Ureña-Cámara

**Affiliations:** 1 Brazilian Army Geographic Service, Brasilia 70630-901, Brazil; 2 Universidad de Jaén, Jaén 23071, Spain

**Keywords:** Geography, Information technology

## Abstract

This article presents datasets prepared with the aim of helping the evaluation of geospatial matching methods for vector data. These datasets were built up from mapping data produced by official Spanish mapping agencies. The testbed supplied encompasses the three geometry types: point, line and area. Initial datasets were submitted to geometric transformations in order to generate synthetic datasets. These transformations represent factors that might influence the performance of geospatial matching methods, like the morphology of linear or areal features, systematic transformations, and random disturbance over initial data. We call our 11 GiB benchmark data ‘MatchingLand’ and we hope it can be useful for the geographic information science research community.

## Background & Summary

Nowadays geospatial data have become ubiquitous in many modern applications. There are a plethora of geospatial data sources generated by different producers that should be integrated in order to provide their full power. We are living a ‘data overload’ scenario, where there are many data providers, each one with its own representation of the geographic reality. Some authors are calling this current status ‘big geospatial data’^1–3^. Another recent trend that can benefit from geospatial data integration is the smart city^4^, which involves the integration of many interdisciplinary fields using geographical knowledge^5^.

Data integration requires finding the correspondences between assessed geospatial datasets, a process that we call geospatial data matching^6^. Data matching is not a trivial task, and one that has demanded recent research from the Geospatial Information Science (GIScience) community^7–10^.

In our previous study^6^ we indicated that the matching research community should create a benchmark dataset for testing new matching methods or measures for geospatial vector data within a homogeneous framework. This matching testbed would undoubtedly be a useful tool for comparing different measures and methods, because we identified that the results may change outside the initial test site. In this context, the main aim of this testbed is to provide a comparing framework for geospatial data matching approaches, which can be useful for the research community or GIS software developers. The testbed supplied is composed of four groups of datasets, of which the first was built from real geospatial data, and it serves as a basis for the other three groups of synthetic data. The testbed can be considered as the data set that allows materializing a design of experiments of methods and measures of matching.

**Figure 1:**
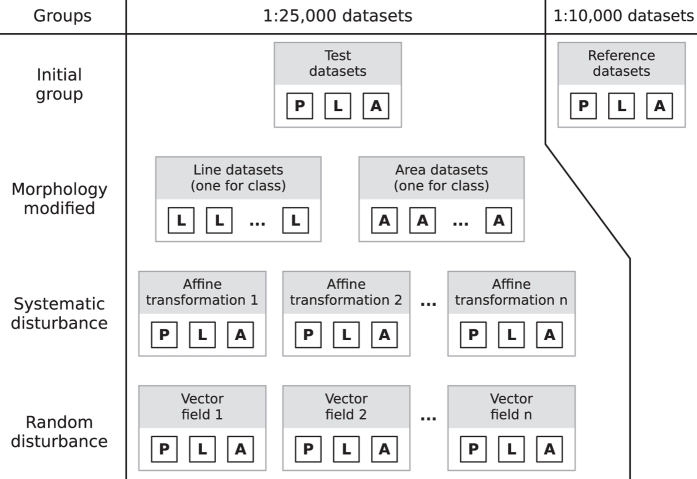
Dataset groups available in this testbed. Each dataset is compounded by one kind of geometry: point (P), line (L) or area (A).

This testbed is composed of four groups of datasets: (1) initial, (2) morphology modified, (3) systematic disturbance, and (4) random disturbance. Initial datasets are originated from authoritative mapping at scales 1:25,000 and 1:10,000 of Spanish agencies. The other three groups are derivative of the initial datasets at scale 1:25,000. Morphology modified datasets are composed of synthetic objects in some morphology class for linear and areal features. Systematic disturbance group is composed of datasets that were generated from affine transformations over initial data. The last group of datasets (random disturbance) is formed by data influenced by displacement vector fields applied over the initial datasets. Each group of datasets is compounded by the datasets for the three geometric primitives: point, line, and area, except for the morphology modified group which does not have point data. Figure 1 illustrates the dataset groups in the matching testbed.

The value of the testbed provided can be summarized in four items: (1) these datasets can be used as benchmark data for other studies investigating geospatial data matching at the feature level; (2) the development of new similarity measures can benefit from these datasets as comparing sets used to calculate the new ‘distances’ between objects; (3) data quality studies focused on positional quality or completeness can use the datasets in order to develop new quality evaluation procedures by adopting two corresponding datasets: one as the test data, and the other as the reference; and (4) there are disturbed data that may permit assessing the robustness of investigated matching techniques in the presence of controlled perturbations.

The remainder of this article is structured as follows: The next section presents the concepts used to produce the datasets. The following subsection explains each data record associated with the testbed supplied, as of the parameters used to generate synthetic data. The procedures that assure the reliability of datasets are discussed in the final section.

## Methods

The geospatial data matching testbed is composed of four groups of datasets: (1) initial, (2) morphology modified, (3) systematic disturbance, and (4) random disturbance. Initial datasets are originated from mapping provided by official mapping agencies of Spain at scales 1:25,000 and 1:10,000. The other three groups are derivative of the initial datasets at scale 1:25,000. Morphology modified datasets are composed of synthetic objects in some morphology class for linear and areal features. Systematic disturbance group is composed of datasets that were generated from affine transformations over initial data. The last group of datasets (random disturbance) is formed by data influenced by displacement vector fields applied over the initial datasets. Each group of datasets is compounded by the datasets for the three geometric primitives: point, line, and area, except for the morphology modified group which does not have point data. Each test dataset (scale 1:25,000) can be divided into nine regions. Each region can be identified by the first number of the object identifier (OID), e.g., 1,023 is in the first region, and 9,128 is in the ninth region.

The following subsections detail the initial datasets and the methods used to prepare each group of datasets.

### Initial datasets

The initial datasets in this testbed are formed by test datasets at scale 1:25,000 and reference datasets at scale 1:10,000. Test data is originated from the Base Topográfica Nacional 1:25,000 (BTN25) of national mapping provided by the Instituto Geográfico Nacional of Spain^11^. Reference data is originated from the Base Cartográfica de Andalucía 1:10,000 (BCA10) of regional mapping provided by the Instituto de Estadística y Cartografia de Andalucía^12^. Test datasets were divided into nine regions (S1-S9) with their corresponding regions in reference datasets (B1-B9).

For our testbed we selected mapping sheets with different landscapes: coast and mountain, rural and urban. Each region was originated from the following mapping sheets 1:25,000: (S1) 0896-3, (S2) 0896-4, (S3) 1003-4, (S4) 0999-1 east, (S5) 0999-2 west and 0999-4 east, (S6) 0999-1 west, (S7) 0999-3, (S8) 0999-4 west, and (S9) 0999-2 east.

The test data was selected from BTN25 as follows: Point data were created from the ‘Building’ class, so the areal features were converted to points using their centroid. First we selected buildings with an area less than 1,000 m^2^ and shape index^13^ less than 1.2. Then for the regions S1-S6 we randomly selected more than 100 objects. For the regions S7-S9 we selected objects from some agglomerated areas in order to be close to an urban environment (more than 100 objects in each area). Area data were also obtained from the Building class, but excluding those objects selected as point data. After excluding point features, we randomly selected more than 100 objects for the regions S1-S6. For the regions S7-S9 we selected some near objects in order to represent an urban environment. In these cases, more than 250 objects were picked in each dataset. For the linear data the first step was to homogenize the road data for the initial datasets (BTN25 and BCA10) using the same topological rules. These procedures avoided ‘broken’ lines and ‘long’ lines. So line data were selected from BTN25 in this order: (1) motorways, (2) roads, (3) links, and (4) tracks. In order to reach at least 125 objects in each region, some tracks were selected randomly.

After these selection procedures over the test data we executed the manual matching between the selected objects from the BTN25 test data against the BCA10 reference data. We used regional orthoimagery to help us in this task in order to dismiss any doubts. At the end of this procedure we had 27 sets (nine regions by three geometries) and their correspondences represented in Table 1.

In Table 1, the column *Geometry* refers to the type of geometry for the dataset. The column *Region* refers to the name of each region. The column *Size* represents the number of objects in each region. The *Matching pairs* columns indicate the number of matching pairs when comparing each test region (S#[PLA]) with each reference region (B#[PLA]). Due to the presence of multiple corresponding case (1:n and m:n) the number of matching pairs differs from the size of the test dataset. For instance, the matching ‘100, 200:101, 102, 103’ represents six matching pairs: 100:101, 100:102, 100:103, 200:101, 200:102, and 200:103.

**Figure 2:**
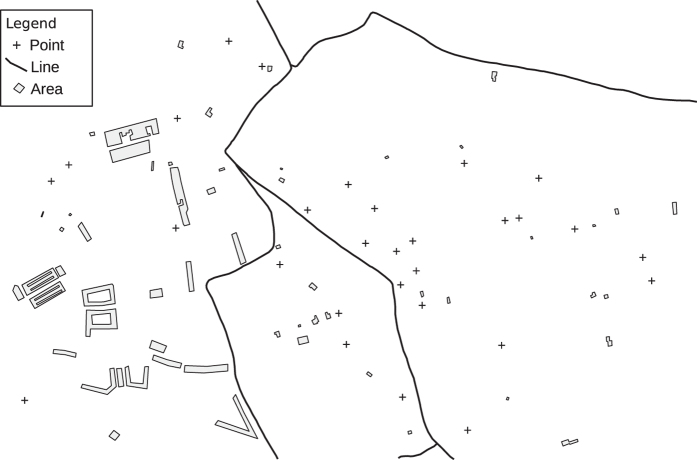
Sample region from MatchingLand.

The last step was translating the areas to a generic place of the world, since they no longer represent any reality. So we also mirrored, rotated or translated the data in order to decharacterize the original site. After that we had all regions in a new compound dataset that we call ‘MatchingLand’ (see Fig. 2).

### Morphology modified

The morphology of linear and areal objects is a factor that may affect the performance of geospatial matching procedures. In order to deal with this factor we adopted a roughness classification for lines and developed a complexity classification for areas. Based on these morphology classifications we developed a method for generating synthetic data from some source data for a specific morphology class.

**Figure 3:**
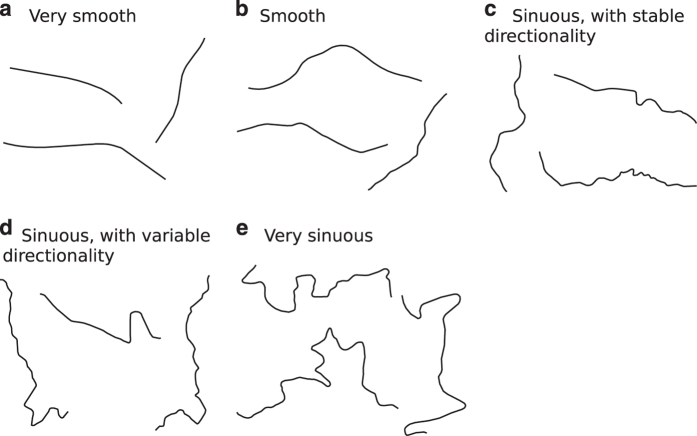
Samples of the morphology classification applied to lines.

The line roughness classification is based on the road-line classification developed by Ariza-López and García-Balboa^14^ where the authors used a back-propagation artificial neural network (BANN) over a moving window. Since we use road data in our experiment, this method seems to be adequate for our purposes. The BANN method defined five established roughness classes for road data: (1) very smooth, (2) smooth, (3) sinuous, with stable directionality, (4) sinuous, with variable directionality, and (5) very sinuous. Figure 3 presents examples of lines classified according to this method.

**Figure 4:**
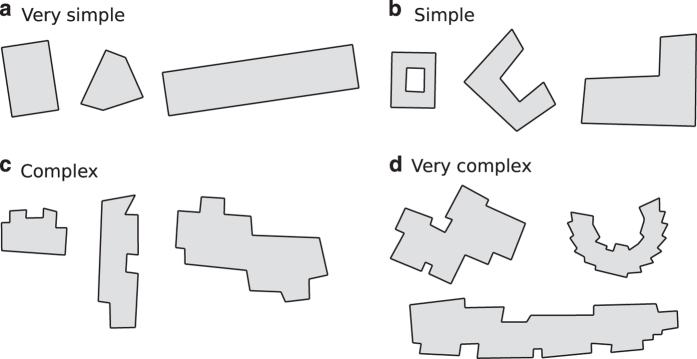
Samples of the morphology classification applied to areas.

The area complexity classification developed in this study is drawn for building data at small scale. This method is based on two concepts: convexedness and Arkin’s turning function^15^. We propose a complexity classification for area building data defined in four classes: (1) very simple, (2) simple, (3) complex, and (4) very complex. Class 1 areas group the simpler objects that are the convex ones without holes. Class 2 areas are those convex ones with holes, and also those objects that are similar to some standard, like ‘L’ or ‘U’ objects. More complex objects are determined according to their number of ‘turns’, i.e., the number of times that the external ring changes its current turning (left or right). Class 3 are those objects with less than or equal to 10 turning changes, while class 4 (very complex) buildings are those that exceed this limit. Figure 4 shows some areas classified according to this method.

With the aim of incrementing the population of lines and areas in each morphology class we propose two methods for generating synthetic data, modifying original sources according to the desired morphology class.

**Figure 5:**
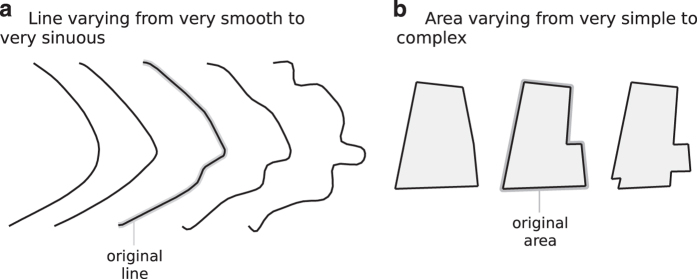
Examples of lines and areas created according to the morphology modified procedure.

The method for lines works as follows: For each line in the original data, we compare its morphology class with the desired morphology class. If the difference is greater than two, or the object already has the desired class, the object remains unaltered. Otherwise, a procedure for smoothing or roughing the line should be applied in order to reach the desired morphology class. The smoothing procedure is a combination of Douglas-Peucker simplification^16^ with Gaussian filtering^17,18^ of sigma 4. The roughing procedure applies random displacements along internal curves of the line (clockwise or counter-clockwise). These procedures do not affect the first or last points of manipulated lines. As these procedures do not take into account the neighbouring objects, some lines required manual edition in order to maintain the topology. Figure 5a shows an example of how a line, originally classified in class 3, can be flattened to class 1 or can be roughened to class 5.

Similar to the line method, the area method also generated synthetic data. The procedure does not affect class 4 areas (very complex). For each area in the original data, we compare its morphology class with the desired morphology class. If there is no difference, the object remains the same. Otherwise, we apply a procedure that randomly disturbs or simplifies the object's geometry in order to achieve the desired morphology class. The disturb procedure raises the complexity of polygons by means of perforating one without holes or creating a 'corner' at a random vertex. The simplify procedure acts over non-convex polygons by removing the corners that least influence the area size. Figure 5b presents an example of an area that in the source data was classified as simple (class 2—'L' shape) that was simplified to class 1 and was disturbed to class 3.

### Systematic disturbance

The presence of positional systematic disturbance is a factor that potentially affects the performance of geospatial data matching procedures. The aim of this group of datasets is to identify the influence of intentional systematic perturbations in position over matching procedures. Moreover, these datasets can also be valuable for data quality research studies that investigate positional quality. Our methodology is similar to the study of Mozas-Calvache and Ariza-López^19^, where the authors simulated several displacements over original data, such as translations, rotations, and scaling.

We propose generating synthetic data from the original data by applying a set of systematic disturbances represented by means of an affine transformation. This transformation is a composition of translations, rotations, and scaling^20^. Hence our systematic disturbance method is designed to reflect these three kinds of transformations. The approach requires a set of standard displacements that define the entire process and it also requires a minimum bounding rectangle (MBR). For each displacement we generate a set of systematic disturbances, that are: (1) translations in eight directions, (2) counter-clockwise and clockwise rotations over three different pivots, and (3) two scaling factors (dilation and shear).

**Figure 6:**
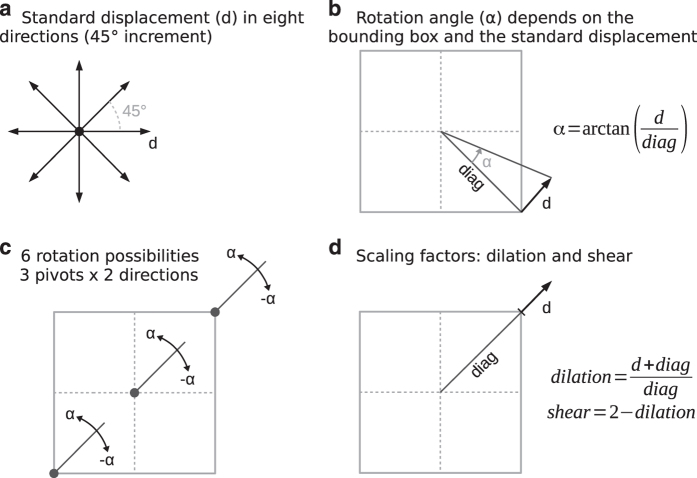
Systematic disturbance in function of a standard displacement. The method combines translations, rotations, and scalings. (**a**) Translations. (**b**) Calculating the rotation angle. (**c**) Rotations. (**d**) Scalings (dilation and shear).

The translations are determined by the standard displacement applied in eight directions, beginning at 0 with increments of 45° (Fig. 6a). The rotation angle is calculated for each dataset, taking into account the standard displacement and the half of MBR's diagonal (Fig. 6b). Using this angle we have six possible rotations: two directions (counter-clockwise and clockwise) by three rotation pivots in relation to the MBR (lower-left, centre, and upper-right) (Fig. 6c). Finally, the scaling factors for dilation and shear are calculated using the relation between half of the MBR's diagonal and the standard displacement, as we can see in Fig. 6d.

After determining the translations, rotations and scalings for each standard displacement, in each dataset (its own MBR), these perturbations must be combined in order to create a set of affine transformations that will be used to generate the synthetic perturbed data. The no-disturb configuration (no translation, no rotation, no scaling) is added prior to creating the affine transformations. Then all possible combinations among translations, rotations and scalings are generated.

For instance, if we choose only two distinct standard displacements, this approach is able to generate more than 1,000 different transformations. The number of combinations is calculated as follows: combinations=translations×rotations×scalings=(1+8×2)×(1+6×2)×(1+2×2)=17×13×5=1,105. For each affine transformation, a new synthetic dataset is created.

### Random disturbance

This last group of datasets acts similarly to the systematic disturbance, but this approach uses random perturbations over original data in order to simulate this random behaviour. Other studies^20^ adopted random errors in each vertex, including correlated displacement by lines. In this study we propose a new methodology to disturb geospatial data using vector fields created for a given standard displacement.

The key-concept of our methodology for random disturbance is the displacement vector field. This vector field works as a ‘force’ field that modifies the geospatial features by acting over their coordinates by means of random displacement.

There are three parameters in this approach: standard displacement, field resolution, and sigma. The standard displacement works as in the systematic disturb (previous subsection), i.e., it defines the amplitude of disturb. The vector field in this method is created according to a regular tesselation of the source data MBR, so we need a field resolution in order to define the cells. Finally, the sigma value represents the internal variation of the standard displacement. For instance, if we use a sigma of 10%, the random displacements will vary ±10% in amplitude in relation to the standard displacement.

**Figure 7:**
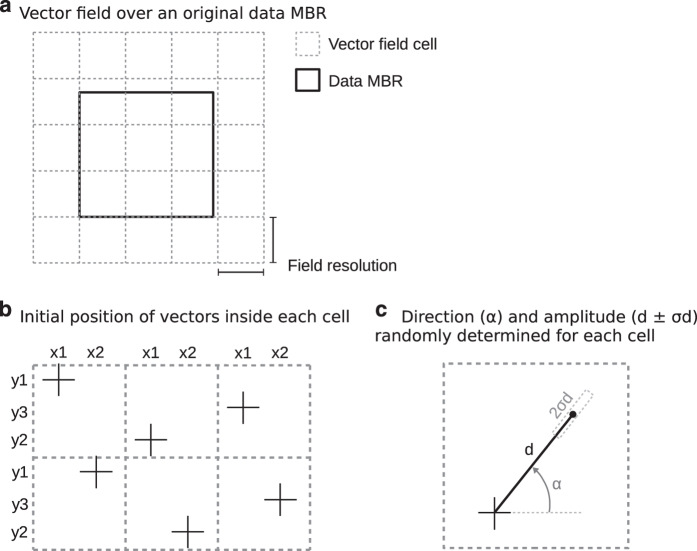
Vector field methodology. (**a**) Tesselation over original data. (**b**) Unaligned position of generators. (**c**) Direction and amplitude are randomly determined.

The vector field disturb method works as follows: After defining the parameters, it creates a regular tesselation using the field resolution over the dataset's MBR. This tesselation has at least two additional columns and rows with the aim being to guarantee that the data border will fit inside the vector field (Fig. 7a illustrates an example). Then, using an unaligned systematic pattern^21^ it randomly creates a set of *x* values (one for each row) and a set of *y* values (one for each column). These values define the coordinates for each generator of our vector field, one per cell (Fig. 7b). The next step is to define the direction and amplitude for each field generator. The direction is randomly determined while the amplitude is calculated in function of the given standard displacement plus a random variation limited by the sigma (σ) parameter (Fig. 7c). In the end we have a vector field with a displacement vector for each cell in the tesselation.

The disturb vector field is composed of a set of displacement vectors which quantify the disturb to be applied in a dataset. We propose using this vector field as a geometric transformation over the original dataset. The influence of each vector in a coordinate of perturbed data should be determined in function of an interpolation function. In this approach we adopt the inverse distance weighting (IDW) interpolation with pow 2 and search radius of 2.5 times the resolution, as indicated by Gumiaux *et al.*^22^. The synthetic perturbed data is generated for each random vector field created from the given parameters (standard displacement, field resolution, and sigma).

### Code availability

TerraLib is an open-source GIS library developed by the Brazilian National Institute for Space Research (INPE)^23^. The AffineGT class in TerraLib is used to create the synthetic data in systematic disturb group. The TerraLib code is licensed under the GNU Lesser General Public License version 2.1 as published by the Free Software Foundation. We have used the TerraLib version 4.2.2 that can be found at the TerraLib repository^24^.

One of the subprojects of TerraLib is TerraOGC—a framework for Web-GIS development that has been used in web services research^25,26^. Inside TerraOGC there is a data quality processing module (DQEval) which contains most of the code related to this testbed. The GeometricTools class contains the methods associated to the morphology modified group (classifiers and transforms). The VectorFieldTransformation class uses the SpatialInterpolation class to generate the data for the random disturbance group. TerraOGC code is licensed under the GNU General Public License version 3. We have used the TerraOGC version 1.2.6 that can be found at its repository^27^.

## Data Records

This section describes the four groups of datasets generated using the methodology described in the previous section. All geoespatial data are supplied in the ESRI Shapefile format^28^. The projection system is UTM zone 28 North with datum WGS-84 (EPSG:32628). The list of matching pairs, composed of object identifiers (OID), is in plain text.

### Initial datasets

The initial group is composed of six datasets: SP, SL, and SA for test data (scale 1:25,000), and BP, BL, and BA for reference data (scale 1:10,000), for point, line and area, respectively. Additionally, this group also contains the list of matching pairs in plain text. Each record is in the form: [OIDt][,OIDt]*:[OIDr][,OIDr]*, where OIDt is the OID in test data, and OIDr is the OID in reference data. For instance, the matching record ‘2009:3203,90386’ means that the object 2009 in test data is corresponding to the objects 3203 and 90386 in reference data.

The datasets are available from *figshare* (Data Citation 1) as a compressed (.zip) file that contains the geospatial data in Shapefile and the matching pairs as plain text (.txt).

### Morphology modified

Using the concepts described in this paper we have generated five synthetic datasets for lines—one for each morphology class. Table 2 presents the object count in the original and modified datasets (CL1-CL5) for lines.

We have generated three synthetic datasets for areas, one for each morphology class except for class 4 (very complex). Table 3 presents the object count in the original and modified datasets (CL1-CL3) for areas.

The datasets are available from *figshare* (Data Citation 1) as a compressed (.zip) file that contains the geospatial data in Shapefile.

### Systematic disturbance

Using the proposed methodology we have elected four standard displacements in order to generate the systematic disturbance: 5, 12.5, 25, and 50 meters. These values were chosen taking into account the Brazilian standard for geospatial data quality^29^. According to this standard, the maximum positional error accepted for 1:25,000 data vary from 7.0 to 25 meters. So we have chosen one value below this limit (5 m), two values inside (12.5 and 25 m), and another value above (50 m). These four standard displacements have originated 7,425 different combinations for each geometry (point, line, and area).

Tables 4,5,6 bring the different configurations for translations, rotations, and scalings (dilation/shear) used to generate the synthetic perturbed datasets. Rotation and scaling transformations depend on the size of diagonal of considered datasets, so the amplitude of these transformations varies for each type of geometry.

Rotations and scalings are considered for each region (S1-S9) in each dataset.

The names of the datasets in this group identify the configuration used by combining the geometry type (SP, SL or SA), translation (t0 to t32), rotation (r0 to r24), and scaling (s0 to s8). For instance, the dataset called ‘SL_t19_r11_s7’ represents test data of line type (SL) which were translated 25 m to the east, rotating −0.00130378 rads using the centre as pivot, and dilating using the factor 1.00522.

The datasets are available from *figshare* (Data Citation 1) as compressed (.zip) files that contain the geospatial data in Shapefile.

### Random disturbance

Using the proposed methodology we chose the same four standard displacements used in systematic disturbance: 5, 12.5, 25, and 50 meters. We adopted a field resolution of 4 km which represents 16 cm in our 1:25,000 test data. The last parameter is the sigma, which defines the internal variation of amplitude. Here we chose a value of ±10%. Hence we randomly generated 100 vector fields for each standard displacement, which results in 400 vector fields. Each vector field configures a geometric transformation that was applied for each type of geometry: point, line, and area.

The names of the datasets in this group identify the configuration used by combining the geometry type (SP, SL or SA), the standard displacement followed by the word ‘random’, and a count the represents the number of the vector field (001 to 100). For instance, the dataset called ‘SA_random12.5_083’ represents test data of area type (SA) which was influenced by a vector field with a standard displacement of 12.5 m, and it is the 83rd field in this configuration.

The datasets are available from *figshare* (Data Citation 1) as compressed (.zip) files that contain the geospatial data in Shapefile.

## Technical Validation

This section presents the analyses performed over MatchingLand testbed in order to assure the technical quality of the datasets supplied. We executed three types of analyses: geospatial data quality, matching pairs checking, and randomization testing.

Geospatial data quality evaluation encompasses the procedures that guarantee the quality of geospatial datasets in Shapefile. We used the quality evaluation framework published in ISO 19157:2013^30^. According to this standard there are five categories of quality elements for geospatial data: completeness, positional accuracy, thematic accuracy, temporal quality, and logical consistency. This testbed is composed essentially of synthetic data, so only the logical consistency can be assessed. In this category we are able to assess two quality elements: format consistency and topological consistency.

We evaluated whether all geospatial datasets stored in the Shapefile format (.shp) were valid against the file format specification^28^. No errors were found for format consistency. We evaluated the topological consistency adopting as the correctness measure the conformance of geometries with the Simple Features Specification^31^ published by Open Geospatial Consortium (OGC). We used the function ST_IsValid from PostGIS^32^ in order to evaluate the geometry conformance. All the geometries assessed passed the test.

The quality of matching pairs supplied throughout this testbed was verified using a sampling scheme described as follows: We adopted the set of object identifiers (OIDs) for each geometry type of test data (1:25,000 scale) as the population to be assessed. For each set of OIDs (point, line, and area) we defined the sampling size using the hypergeometric distribution^33^ and an expected quality (correctness) of 95%. Hence we randomly selected 69 objects in point data (population equal to 1,215 objects), 70 objects in line data (population 1,255), and 71 objects in area data (population 1,686). These samples of matching pairs were verified by GIS analysts distinct from those that matched the pairs manually. The manual verification found discrepancies below 5%.

The last quality analysis refers to the randomness of displacement vector fields used to create the random disturb group of datasets. We used the variogram approach^34^ to evaluate any bias in vector fields by taking a look into the spatial dependence of their vectors. Using the geoR package^35^ we identified that there is no spatial dependence in the displacement vector fields.

## Usage Notes

The datasets of MatchingLand are designed to test geospatial data matching methods by using geometric, topologic, or context measures. However, these datasets can also be used to test quality evaluation methods, by adopting one dataset as test data, and their corresponding dataset as the reference data. In this section we present some examples of how to use these datasets in matching or data quality research.

In the first use case let’s consider a study of a new matching method for linear data, like a recent study^36^. The researcher can take the dataset SL (initial group, test) and the dataset BL (initial group, reference), apply his matching method over these datasets, and then compare the generated matching pairs with the rule of thumb provided along MatchingLand. So it is possible to calculate some performance parameters, like the precision and recall^37^, and then compare the new method with others using the same datasets.

The researcher can also verify whether his method is sensible to variations in the line morphology. In this case he can take the datasets of the morphology modified group (line datasets) and execute the matching against the reference data (dataset BL). The performance can be assessed in the same way of the initial group, using precision and recall measures. It allows to evaluate whether the new method is sensible to the line morphology.

Continuing with his experiment, the researcher can determine if his method is sensible to systematic displacements, more specifically translations. In order to do this he can take the systematic disturbance line datasets in which occurs only translations: SL_t*α*_r0_s0, *α*∈[1, 32], and matching them against the reference dataset (BL). Once more, the generated matching pairs can be compared with the rule of thumb to permit a precision/recall comparison. Similar procedure should be applied in order to determine whether the new method is robust in presence of random disturbance, with different amplitudes. In this case the researcher should take datasets from the random disturbance group (SL_random*A*_*β*, *A* is the amplitude and *β*∈[1,100]), match them against the reference dataset (BL), and compare the results using precision/recall.

In other use case, a researcher is studying a new quality evaluation procedure for positional accuracy assessment using a point-based method, like the NMAS^38^ or the NSSDA^39^. In this case he can evaluate the point test dataset (SP) against the point reference dataset (BP) using the homologous points, since the matching pairs are provided along the datasets in MatchingLand. The researcher can apply his own restrictions, like using only 1:1 pairs in this assessment. Other possibility is to test different sampling schemes, since there are more than 1,000 1:1 pairs in the initial group for points (see Table 1).

The datasets available in MatchingLand can be divided into nine regions, which can be interesting whether the researcher is working with design of experiment techniques^33^. The examples explained in this section intend to be illustrative, but not exhaustive. More examples of how to use the datasets of MatchingLand to test matching methods or quality procedures can be found in this study^40^.

## Additional information

**How to cite this article:** Xavier, E. M. A. *et al.* MatchingLand, geospatial data testbed for the assessment of matching methods. *Sci. Data* 4:170180 doi: 10.1038/sdata.2017.180 (2017).

**Publisher’s note:** Springer Nature remains neutral with regard to jurisdictional claims in published maps and institutional affiliations.

## Supplementary Material



## Figures and Tables

**Table 1:** Regions considered in the initial dataset group and their sizes.

			Matching pairs		
Geometry	Region	Size	1:1	1:n	m:n
Point	S1P	112	91	2	0
	S2P	115	100	6	0
	S3P	126	115	2	0
	S4P	119	98	4	0
	S5P	120	108	2	0
	S6P	120	94	4	0
	S7P	184	170	6	0
	S8P	201	188	0	0
	S9P	118	106	18	0
Line	S1L	133	50	158	29
	S2L	135	61	148	14
	S3L	140	67	128	34
	S4L	138	51	160	64
	S5L	135	37	109	92
	S6L	140	30	233	89
	S7L	140	57	120	54
	S8L	143	56	56	153
	S9L	151	87	93	0
Area	S1A	123	101	19	0
	S2A	140	87	81	0
	S3A	140	122	18	0
	S4A	150	120	33	0
	S5A	202	172	63	0
	S6A	160	107	44	0
	S7A	268	215	55	0
	S8A	253	202	61	8
	S9A	250	159	130	59

**Table 2:** Number of objects in each dataset according to the line morphology classification.

Morphology class	Dataset					
	Original	CL1	CL2	CL3	CL4	CL5
1	909	1206	564	571	909	909
2	193	8	642	49	28	193
3	112	0	37	626	165	16
4	29	29	0	8	152	96
5	12	12	12	1	1	41

**Table 3:** Number of objects in each dataset according to the area morphology classification.

Morphology class	Dataset			
	Original	CL1	CL2	CL3
1	1108	1630	1	0
2	202	0	1630	0
3	325	5	4	1635
4	51	51	51	51

**Table 4:** Translations generated for the four standard displacements.

Configuration	Translation in X (m)	Translation in Y (m)	Configuration	Translation in X (m)	Translation in Y (m)
t0	0	0			
t1	5	0	t17	25	0
t2	3.53553	3.53553	t18	17.6777	17.6777
t3	0	5	t19	0	25
t4	−3.53553	3.53553	t20	−17.6777	17.6777
t5	−5	0	t21	−25	0
t6	−3.53553	−3.53553	t22	−17.6777	−17.6777
t7	0	−5	t23	0	−25
t8	3.53553	−3.53553	t24	17.6777	−17.6777
t9	12.5	0	t25	50	0
t10	8.83883	8.83883	t26	35.3553	35.3553
t11	0	12.5	t27	0	50
t12	−8.83883	8.83883	t28	−35.3553	35.3553
t13	−12.5	0	t29	−50	0
t14	−8.83883	−8.83883	t30	−35.3553	−35.3553
t15	0	−12.5	t31	0	−50
t16	8.83883	−8.83883	t32	35.3553	−35.3553

**Table 5:** Rotations generated for the four standard displacements.

Configuration	Pivot	Angle for Point data (rad)	Angle for Line data (rad)	Angle for Area data (rad)
r0	lower left	0	0	0
r1	lower left	0.000591397	0.000521513	0.000574903
r2	centre	0.000591397	0.000521513	0.000574903
r3	upper right	0.000591397	0.000521513	0.000574903
r4	lower left	−0.000591397	−0.000521513	−0.000574903
r5	centre	−0.000591397	−0.000521513	−0.000574903
r6	upper right	−0.000591397	−0.000521513	−0.000574903
r7	lower left	0.00147849	0.00130378	0.00143726
r8	centre	0.00147849	0.00130378	0.00143726
r9	upper right	0.00147849	0.00130378	0.00143726
r10	lower left	−0.00147849	−0.00130378	−0.00143726
r11	centre	−0.00147849	−0.00130378	−0.00143726
r12	upper right	−0.00147849	−0.00130378	−0.00143726
r13	lower left	0.00295698	0.00260756	0.00287451
r14	centre	0.00295698	0.00260756	0.00287451
r15	upper right	0.00295698	0.00260756	0.00287451
r16	lower left	−0.00295698	−0.00260756	−0.00287451
r17	centre	−0.00295698	−0.00260756	−0.00287451
r18	upper right	−0.00295698	−0.00260756	−0.00287451
r19	lower left	0.0059139	0.00521509	0.00574897
r20	centre	0.0059139	0.00521509	0.00574897
r21	upper right	0.0059139	0.00521509	0.00574897
r22	lower left	−0.0059139	−0.00521509	−0.00574897
r23	centre	−0.0059139	−0.00521509	−0.00574897
r24	upper right	−0.0059139	−0.00521509	−0.00574897
There are distinct angle values for each type of geometry.				

**Table 6:** Scalings generated for the four standard displacements.

Configuration	Scaling factor for Point data	Scaling factor for Line data	Scaling factor for Area data
s0	1	1	1
s1	1.00059	1.00052	1.00057
s2	0.999409	0.999478	0.999425
s3	1.00148	1.0013	1.00144
s4	0.998522	0.998696	0.998563
s5	1.00296	1.00261	1.00287
s6	0.997043	0.997392	0.997125
s7	1.00591	1.00522	1.00575
s8	0.994086	0.994785	0.994251
There are distinct values of scaling factor for each type of geometry.			
